# Schiff base-assisted catalytic fabrication of TiO_2_ nanoparticles for antibacterial applications against multidrug-resistant strains

**DOI:** 10.1128/spectrum.02641-25

**Published:** 2026-05-22

**Authors:** Farasat Bibi, Ahtaram Bibi, Naila Khalid, Nazia Sultan, Mubbashir Hussain, Sandal Wafa, Abdul Rehman, Niaz Muhammad

**Affiliations:** 1 Department of Microbiology, Kohat University of Science and Technology 66860, Kohat, Pakistan; 2 Department of Chemistry, Kohat University of Science and Technology 66860, Kohat, Pakistan; Seton Hall University, South Orange, New Jersey, USA

**Keywords:** multidrug-resistant bacteria, antibacterial activity, titanium dioxide (TiO_2_) nanoparticles

## Abstract

The emergence of multidrug-resistant (MDR) bacteria poses a significant global health challenge, necessitating the development of novel antimicrobial agents. In this study, titanium dioxide (TiO_2_) nanoparticles were synthesized via a sol-gel method using Schiff base (azomethine) complexes as catalytic ligands. Structural and morphological characterization was performed using UV–visible spectroscopy, FTIR, SEM, and XRD, confirming the formation of anatase-phase TiO_2_ nanoparticles with average sizes around 100 nm. The incorporation of the azomethine ligand was verified by characteristic functional group shifts and spectral absorption in the UV region. Antibacterial activity was assessed against MDR strains of *Escherichia coli*, *Staphylococcus aureus*, and *Klebsiella* spp., using the well diffusion method. Results showed that both the azomethine compound (C2) and TiO_2_ nanoparticles (NP2 and NP3) exhibited significant antibacterial effects, with NP2 demonstrating the largest zones of inhibition. MIC and MBC assays further confirmed the superior potency of NP2, showing the lowest inhibitory and bactericidal concentrations, particularly against *S. aureus*. These findings highlight the potential of Schiff base-modified TiO_2_ nanoparticles as effective antimicrobial agents against resistant pathogens. The study offers a promising route toward the development of nanomaterial-based alternatives to conventional antibiotics.

**IMPORTANCE:**

This study addresses the global health crisis caused by “superbugs” bacteria that have become resistant to multiple standard antibiotics. Traditional treatments are failing, creating an urgent need for new ways to fight infections. Researchers developed a novel solution by creating microscopic titanium dioxide particles enhanced with a chemical “template” called a Schiff base. The significance of this work lies in its potential to provide a powerful alternative to traditional medicine. These enhanced nanoparticles proved highly effective at killing dangerous resistant bacteria like *Escherichia coli* and Staph by disrupting their protective outer layers. Because they use multiple physical mechanisms to attack, it is much harder for bacteria to develop resistance to them. This research offers a promising, eco-friendly roadmap for developing next-generation antimicrobial treatments.

## INTRODUCTION

The increasing prevalence of multidrug-resistant (MDR) bacterial infections has emerged as one of the most pressing global health concerns of the 21st century. The overuse and inappropriate prescription of antibiotics in both human healthcare and livestock industries have significantly accelerated the evolution of antibiotic-resistant strains (1). According to estimates from the Centers for Disease Control and Prevention (CDC), antibiotic-resistant bacteria are responsible for over 3 million serious infections and approximately 25,000 deaths annually in the United States alone (2). This resistance typically arises either through inherent bacterial traits or via the acquisition of mutations following repeated exposure to antibiotics (3). As conventional antimicrobial treatments become increasingly ineffective, there is a growing demand for alternative therapeutic strategies that can effectively combat resistant pathogens while minimizing side effects and environmental impact.

Nanotechnology has emerged as a promising avenue in this context, offering materials with novel functionalities due to their nanoscale dimensions. Among various metal oxide nanomaterials, titanium dioxide (TiO_2_) nanoparticles stand out owing to their high surface-area-to-volume ratio, strong oxidative potential, photostability, and low toxicity (4, 5). These properties make TiO_2_ a suitable candidate for diverse applications, including photocatalysis, water purification, biosensing, and antimicrobial formulations. More importantly, TiO_2_ nanoparticles exhibit intrinsic antibacterial activity, which can be further enhanced when conjugated with conventional antibiotics or synthesized in combination with other biologically active compounds (6). Such synergistic effects disrupt bacterial membranes through multiple mechanisms, decreasing the likelihood of resistance development. A particularly promising route for the functionalization and controlled synthesis of TiO_2_ nanoparticles involves the use of Schiff base ligands. Schiff bases, containing the characteristic azomethine group (–C = N–), are known for their excellent chelating ability and structural versatility. These compounds exhibit a wide spectrum of biological activities, including antimicrobial, anticancer, antimalarial, antiviral, and anti-inflammatory effects (7). The presence of heteroatoms and conjugated systems in Schiff bases enhances their reactivity and coordination behavior, making them valuable in the design of metal complexes with improved biological and catalytic performance.

When complexed with transition metals such as titanium, Schiff bases can act as tailored precursors for nanoparticle synthesis, enabling control over size, morphology, and surface properties of the resulting TiO_2_ nanomaterials. Such titanium Schiff base complexes have been reported to influence the nucleation and growth of TiO_2_ nanoparticles under controlled thermal or catalytic conditions (8). Furthermore, azole-based Schiff base derivatives are employed industrially in dye and textile applications, underscoring their chemical robustness and multifunctionality.

Despite extensive research into titanium dioxide (TiO_2_) nanoparticles for antimicrobial use, their full potential against multidrug-resistant (MDR) bacteria remains limited by inefficient synthesis methods, poor stability, and low bioavailability. Existing studies rarely explore Schiff base complexes as catalytic agents for nanoparticle fabrication, which could enhance antibacterial efficacy through improved surface reactivity and ligand-mediated targeting. Furthermore, the synergistic role of Schiff bases in optimizing TiO_2_ nanoparticle morphology and bactericidal activity remains underexplored. This study addresses this critical gap by investigating Schiff base-assisted catalytic synthesis of TiO_2_ NPs and evaluating their enhanced antibacterial activity against clinically relevant MDR pathogens. So, this study explores the catalytic synthesis of TiO_2_ nanoparticles using titanium-Schiff base complexes as molecular templates. It aims to investigate the structural, morphological, and antimicrobial characteristics of the resulting nanoparticles, particularly against MDR bacterial strains. By combining the biochemical versatility of Schiff bases with the known photocatalytic and antimicrobial properties of TiO₂, this research contributes to the development of innovative nanomaterials for combating antibiotic resistance.

## MATERIALS AND METHODS

The reagents used in this study were p-aminophenol, terephthalaldehyde (TPA), absolute ethanol, glacial acetic acid, n-hexane, ethyl acetate, titania (TiO_2_), distilled water, and dimethyl sulfoxide (DMSO), all analytical grade and used without further purification.

### Synthesis of Schiff base, N-(p-hydroxyphenylimino)methyl)benzylidene-4-hydroxybenzenamine

An ethanolic solution (50 mL, 4.5 mM, 2.23 mmol) of terephthalaldehyde (TPA) and a few drops of glacial acetic acid was taken in a two-neck round-bottom flask, equipped with a condenser and drying tube. p-Aminophenol (0.6 g, 5.5 mmol) was added, stirred well, and heated to reflux for 6 h until the formation of the orange-brown precipitate. The reaction was monitored by thin layer chromatographic plates (E-Merck 60 F254), using the following solvent system: n-hexane:ethyl acetate (3:2). After completion of the reaction, the Schiff base was vacuum filtered, washed thoroughly with plenty of water, dried, and recrystallized from ethanol, obtaining a yellow powder. Color: orange brown, % yield: 87%, mp: 275°C (Lit mp 274°C).

### Synthesis of titanium dioxide nanoparticles

In a 250 mL round-bottom flask, 0.1 g of titanium dioxide was dissolved in 50 mL of ethanol and stirred for 30 min. A few drops of distilled water were added to create a dispersion medium, followed by ultrasonication for 30 min. The mixture was transferred to an autoclave and heated at 121°C for 2 h. After cooling, the solution was washed and centrifuged with deionized water to remove impurities (4). In addition to the Schiff base-assisted synthesis, a control sample of TiO_2_ nanoparticles was prepared under identical experimental conditions without the addition of the Schiff base ligand. This control sample is designated as NP3 and was used as a reference to evaluate the influence of the Schiff base on the structural properties and antibacterial activity of the synthesized nanoparticles.

### Schiff base-assisted catalytic fabrication of TiO_2_ nanoparticles

A 250 mL two-neck round-bottom flask was charged with 0.5 g Schiff base, N-(p-hydroxyphenylimino)methyl) benzylidene-4-hydroxybenzenamine, added 100 mL of ethanol, stirred well, and then added 10 mL of deionized water to form a dispersion medium. The reaction mixture was sonicated for 40 min, followed by the addition of dried TiO_2_ nanoparticles and stirred well for 3 h. The color change from dark brown to light purple confirmed the titanium dioxide nanoparticle-Schiff base complex formation. The final product was filtered and dried at 500°C for 2 h.

Characterization of the nanoparticles was conducted using a combination of analytical techniques. Scanning electron microscopy (SEM) was used to assess surface morphology and particle distribution. X-ray diffraction (XRD) provided insights into the crystalline structure, while Fourier transform infrared spectroscopy (FTIR) using a Bruker Alpha-P instrument confirmed functional group bonding. UV–visible spectroscopy was performed to assess optical absorption properties, relevant for understanding the material’s interaction with bacterial cell membranes (9). For comparative analysis, TiO_2_ nanoparticles synthesized in the presence of the Schiff base ligand were designated as NP2, whereas TiO_2_ nanoparticles synthesized without the Schiff base under identical conditions were labeled as NP3 and served as the control sample.

The antibacterial activity of the synthesized azomethine compound and TiO_2_ nanoparticles was evaluated against clinically relevant multidrug-resistant (MDR) strains, including *Escherichia coli*, *Staphylococcus aureus*, and *K. pneumoniae* spp., using the well diffusion method in triplicate (*n* = 3) (10). Mueller-Hinton agar (MHA) was prepared and autoclaved at 121°C for 1 h before being poured into sterile petri dishes. Overnight bacterial cultures were uniformly swabbed across the plates, and wells (6.25 mm diameter) were loaded with 100 µg/mL of either the azomethine compound (C2) or TiO_2_ nanoparticles (NP2) dissolved in DMSO. In the antibacterial assay, dimethyl sulfoxide (DMSO) was used as the negative control to confirm that the solvent itself did not produce antibacterial effects, while commercially available antibiotic discs were used as positive controls to validate the susceptibility of the tested bacterial strains. The plates were incubated at 37°C for 24 h, and zones of inhibition were measured to assess antibacterial efficacy.

Minimum inhibitory concentration (MIC) and minimum bactericidal concentration (MBC) were determined using standard broth microdilution protocols in sterile 96-well plates, following Clinical and Laboratory Standards Institute (CLSI) guidelines. Serial twofold dilutions of TiO_2_ nanoparticles were prepared in Mueller-Hinton Broth (MHB), and bacterial inoculum (1–2 × 10⁶ CFU/mL) was added. Plates were incubated at 37°C for 24 h, and MIC was recorded as the lowest concentration without visible growth. For MBC determination, 10 µL aliquots from non-turbid wells were plated onto Tryptic Soy Agar (TSA) and incubated. The lowest concentration showing no colony growth was recorded as the MBC.

This integrated approach aimed to synthesize and evaluate azomethine-functionalized TiO_2_ nanoparticles as potential agents against MDR bacteria by leveraging sol-gel catalysis, structural characterization, and biological assessment techniques.

## RESULTS AND DISCUSSION

**Fig 1:**
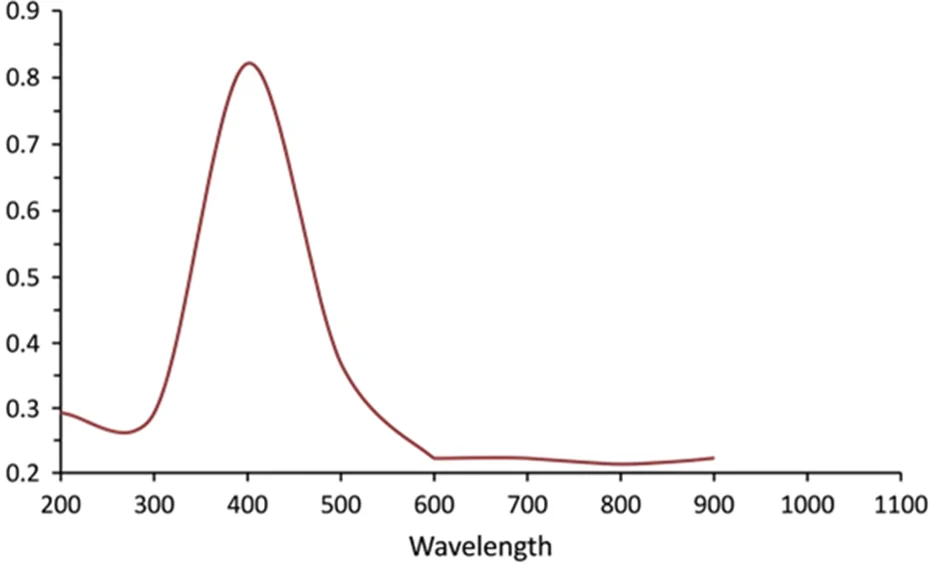
UV–visible spectrum of TiO_2_ sharp edge at 400–430 nm showing that TiO_2_ nps absorb light from the ultraviolet region at room temperature displaying a good absorption band in the UV region.

UV–visible spectroscopy analysis (Fig. 1) of TiO_2_ nanoparticles was as follows. The UV–visible (UV–Vis) absorption spectrum of the synthesized titanium dioxide (TiO_2_) nanoparticles exhibited a prominent absorption edge between 400 and 430 nm with a maximum absorbance of approximately 0.1. This indicates that the nanoparticles demonstrate significant absorption in the ultraviolet region, specifically in the near-UV range. An absorbance value below 1 suggested low optical density, which typically correlates with well-dispersed, non-aggregated nanoparticles in solution. The sharp absorption edge at ~400–430 nm is characteristic of the electronic transition between the valence band and conduction band of TiO_2_, indicating the material’s semiconducting nature (Fig. 1). The observed absorption behavior aligns with the known optical properties of anatase-phase TiO_2_ nanoparticles, which typically exhibit absorption in the UV region due to their wide bandgap energy (~3.2 eV) (11). The absorption in this region is attributed to charge-transfer transitions from the oxygen 2p valence band to the titanium 3d conduction band. The sharpness of the absorption edge also suggests a relatively uniform size distribution and high crystallinity of the nanoparticles, as broader peaks are often associated with polydispersity and defect states.

The absorption peak of TiO_2_ nanoparticles is commonly reported within the 380–430 nm range, depending on factors such as particle size, phase composition (anatase, rutile, and brookite), surface modifications, and synthesis methods. For instance, Wang et al. (9) and Javed et al. (4) reported TiO_2_ nanoparticle absorption peaks in the 390–420 nm range, consistent with anatase-phase particles synthesized via sol-gel and hydrothermal methods. The absorption range observed in this study is therefore in agreement with prior literature, confirming successful synthesis and UV-light activity of the nanoparticles (Fig. 1).

In addition, the fact that the absorbance remained below 1 in the UV range without significant background scattering suggests that the nanoparticles are well-dispersed and not excessively aggregated, which is a desirable property for further photocatalytic or antibacterial applications (9). The UV absorption capability of TiO_2_ is a key factor contributing to its photocatalytic and antimicrobial activity, as the absorption of UV light leads to the generation of reactive oxygen species (ROS), such as hydroxyl radicals and superoxide ions, which can damage bacterial cell membranes (10). Hence, the UV–visible spectral results support the potential use of these TiO_2_ nanoparticles in antimicrobial applications, especially under UV-light activation. The UV–visible spectrum of the synthesized TiO_2_ nanoparticles revealed a sharp absorption edge at 400–430 nm, consistent with the bandgap characteristics of anatase-phase TiO_2_. This confirms their capability to absorb in the UV region and supports their potential utility in photocatalytic and antibacterial applications. The results are in close agreement with earlier findings, validating the synthesis approach and structural integrity of the nanoparticles.

**Fig 2:**
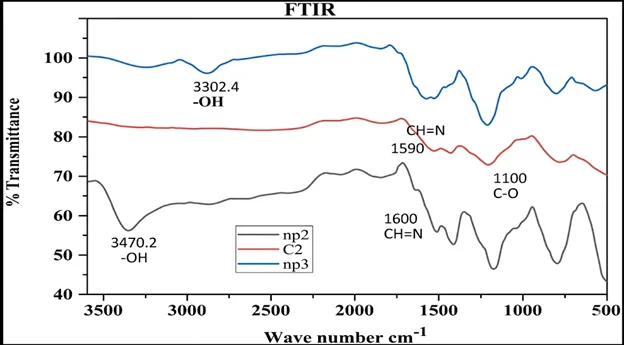
FTIR spectra showing the functional groups in NP2, NP3, and C2. Peaks indicating O-H, CH = O, CH = N, and Ti-O bonds are highlighted to demonstrate the presence and stabilization of TiO_2_ nanoparticles.

The FTIR spectrum provided in Fig. 2 presents a comparative analysis of three samples: the azomethine compound (C2), and two synthesized azomethine-based TiO_2_ nanoparticles (NP2 and NP3). This spectrum helps confirm the presence of specific functional groups and interactions between the organic Schiff base (azomethine) ligand and the TiO_2_ nanoparticles.

The FTIR spectrum (Fig. 2) displays the following key absorption bands. In hydroxyl (–OH) stretching, a broad absorption band is observed at 3,470.2 cm⁻¹ for NP2 and 3,302.4 cm⁻¹ for NP3, indicating the presence of hydroxyl groups. This broadening is typical for –OH stretching vibrations due to hydrogen bonding, often associated with adsorbed water or surface hydroxyls on TiO_2_ nanoparticles (12). The shift to a slightly lower wavenumber in NP3 suggests a difference in surface hydration or degree of interaction with the organic matrix.

In azomethine (C = N) stretching, a prominent peak appears around 1,600 cm⁻¹ in NP2 and 1,590 cm⁻¹ in C2, which corresponds to the stretching vibration of the azomethine (CH = N) group. The presence of this peak in both the pure Schiff base (C2) and the nanoparticle composites (NP2 and NP3) confirms the retention of the azomethine functionality after TiO_2_ incorporation. The slight shift from 1,590 cm⁻¹ (C2) to 1,600 cm⁻¹ (NP2) suggests coordination of the azomethine nitrogen to the titanium center, indicating successful complexation and interaction with TiO_2_ (8).

In C-O stretching, a distinct peak at 1,100 cm⁻¹ in the spectrum of C2 is attributed to C-O stretching vibrations, typically arising from phenolic or ether groups present in the Schiff base structure. This band is less prominent in the TiO_2_ nanocomposites (NP2 and NP3), suggesting possible involvement of the C–O group in bonding or interaction with the TiO_2_ surface (Fig. 2).

The spectral features observed are consistent with previous studies on metal–Schiff base complexes and TiO_2_-based nanocomposites. According to Raczuk et al. (8), Schiff bases display strong azomethine (C = N) stretching around 1,590–1,620 cm⁻¹, and coordination to metal ions often causes a shift in this band due to electron donation from the imine nitrogen to the metal center. The observed shift in NP2 aligns with this coordination behavior, confirming the formation of a Ti–N bond. Similarly, the broad –OH band in the 3,300–3,500 cm⁻¹ range has been widely reported in literature as a characteristic of surface hydroxyl groups in sol-gel-synthesized TiO_2_ nanoparticles (4). These surface –OH groups are important for catalytic and antibacterial properties, as they can facilitate surface reactivity and generation of reactive oxygen species (ROS).

The absence or weakening of certain organic peaks (like C-O) in the nanocomposite spectra may indicate successful integration of the Schiff base with the TiO_2_ matrix, possibly through covalent bonding or surface adsorption. This is in agreement with previous findings that TiO_2_ functionalized with organic ligands exhibits shifts and intensity changes in FTIR spectra due to metal–ligand interactions (9). In conclusion, the FTIR spectra confirm the successful synthesis of azomethine-based TiO_2_ nanoparticles (NP2 and NP3). The presence and shifting of characteristic peaks such as CH = N and –OH demonstrate effective incorporation and interaction of the Schiff base with the TiO_2_ framework. These findings are in strong agreement with prior reports and support the structural and functional integrity of the synthesized nanocomposites for further applications such as antibacterial testing and photocatalysis.

### SEM analysis of TiO_2_ nanoparticles

**Fig 3:**
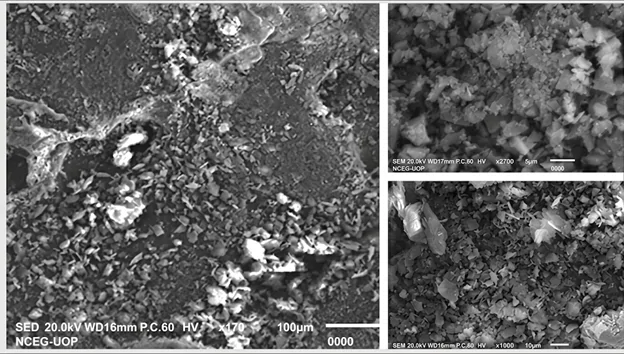
SEM of TiO_2_ nanoparticles of 100 nm at a magnification of 10,000×.

The surface morphology and particle distribution of the synthesized titanium dioxide (TiO_2_) nanoparticles (Fig. 3) were characterized using scanning electron microscopy (SEM). The SEM micrograph revealed that the TiO_2_ nanoparticles were predominantly monodispersed with irregular shapes, and the average particle size was estimated to be approximately 100 nm at a magnification of 10,000×. This nanoscale dimension indicates successful formation of nanoparticles through the sol-gel method. The relatively uniform dispersion also suggests effective control over agglomeration during synthesis.

The presence of irregularly shaped yet evenly distributed nanoparticles may be attributed to the role of the azomethine compound, which likely served not only as a surface-modifying agent but also as a mild capping or stabilizing agent during the nucleation and growth phases of TiO_2_ nanoparticle formation (Fig. 3). This observation supports the hypothesis that azomethine compounds can participate in regulating the crystallization process, possibly by interacting through their functional groups (imine nitrogen or phenolic hydroxyls) with the forming Ti precursors, thus influencing particle shape and size (12).

These morphological features align with earlier findings reported by Javed et al. (4), where Schiff base-modified metal oxide nanoparticles exhibited monodispersity and nanoscale morphology. Similarly, research by Wang et al. (9) documented that TiO_2_ nanoparticles synthesized using organic stabilizers often display irregular but controlled particle morphology, which contributes to enhanced surface area and reactivity. The particle size in the current study (≈100 nm) also falls within the range considered optimal for photocatalytic and antimicrobial applications, as reported in multiple studies.

Additionally, the lack of significant agglomeration in the SEM image indicates that the synthesis parameters, particularly the use of sonication, appropriate solvent ratios, and azomethine incorporation, were effective in preventing particle clumping, a common issue in nanoparticle synthesis. The SEM analysis confirmed the successful synthesis of monodispersed, irregularly shaped TiO_2_ nanoparticles with an average particle size of ~100 nm. The azomethine compound played a significant role in controlling morphology, supporting its catalytic and stabilizing potential in nanoparticle synthesis. These observations are consistent with previous reports on Schiff base-modified metal oxides and reinforce the suitability of the synthesized TiO_2_ nanoparticles for subsequent applications such as antimicrobial testing and photocatalytic performance.

### XRD analysis of TiO_2_ nanoparticles

**Fig 4:**
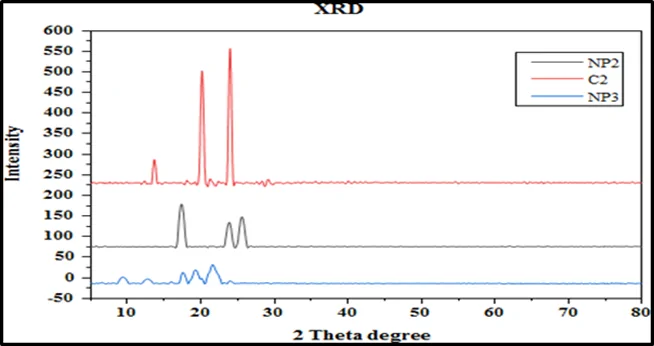
X-ray diffraction (XRD) patterns of titanium nanoparticles NP2, NP3, and C2, showing the crystalline phases and peak positions indicative of the anatase structure. The figure highlights the prominent peaks at 2θ = 25.4°, 25°, and 48°, confirming the presence of the anatase phase and indicating the size and purity of the nanoparticles.

The X-ray diffraction (XRD) analysis (Fig. 4) was employed to determine the crystalline structure and phase composition of the synthesized compounds and nanoparticles namely C2 (azomethine compound), NP2 (azomethine-based TiO_2_ nanoparticles), and NP3 (TiO_2_ nanoparticles without azomethine). The diffractogram clearly shows differences in crystallinity and phase characteristics across the three samples. The XRD pattern of C2 (red line) exhibits sharp and intense peaks predominantly in the range of 2θ = 20–30°, indicating the crystalline nature of the pure azomethine compound (Fig. 4). The presence of these distinct diffraction peaks suggests that the compound forms a well-ordered lattice structure. This is consistent with previous studies where Schiff base derivatives were reported to crystallize in highly ordered phases due to extensive π-conjugation and intermolecular hydrogen bonding.

In contrast, the diffractogram of NP2 (black line), representing TiO_2_ nanoparticles synthesized with the azomethine compound, shows broader and less intense peaks within the same 2θ range (notably near 25°, 27°, and 31°), which are characteristic of the anatase phase of TiO_2_. The broadness of the peaks is indicative of nanocrystalline nature and small crystallite size, confirming successful synthesis of TiO_2_ in nanoscale form (Fig. 4). Moreover, the reduced intensity compared to the C2 pattern reflects partial loss of long-range order due to the incorporation of TiO_2_ and nanoparticle formation. The NP3 sample (blue line), which represents TiO_2_ nanoparticles synthesized without azomethine, displays an even lower intensity and broader peaks than NP2, suggesting lower crystallinity and more amorphous behavior. This comparison indicates that the presence of the azomethine compound (C2) enhanced crystallinity during nanoparticle synthesis, likely due to its role as a templating or chelating agent that facilitated better ordering of TiO_2_ nanocrystals.

These findings are in alignment with earlier reports, where the sol-gel synthesis of TiO_2_ nanoparticles in the presence of organic compounds such as Schiff bases resulted in enhanced phase purity and control over crystallite morphology. Furthermore, the characteristic anatase TiO_2_ diffraction peaks around 25° (101), 27° (110), and 31° (004) agree with standard JCPDS card no. 21-1272, confirming the formation of the anatase phase, which is highly desirable due to its excellent photocatalytic and antimicrobial properties. The XRD results confirm that the azomethine compound (C2) is crystalline, while the TiO_2_ nanoparticles (NP2 and NP3) exhibit nanocrystalline and partially amorphous behavior. The incorporation of the azomethine compound during TiO_2_ nanoparticle synthesis (in NP2) enhanced the crystallinity and phase stability compared to NP3. These results are consistent with previous literature and support the hypothesis that azomethine derivatives play a critical role in directing the crystallization and structural integrity of metal oxide nanoparticles.

### Antibacterial activity of azomethine compound (C2)

**Fig 5:**
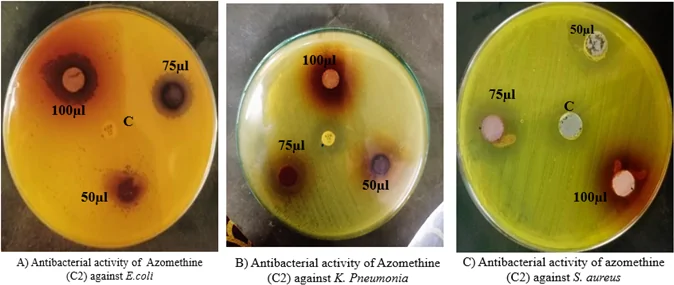
Antibacterial activity of azomethine compound (C2) against (**A**) *E. coli*, (**B**) *K. pneumonia*, and (**C**) *S. aureus.*

The synthesized azomethine compound (C2) demonstrated significant antibacterial activity against multidrug-resistant (MDR) bacterial strains, including *E. coli*, *S. aureus*, and *K. pneumoniae* (Fig. 5A through C and Table 1). The antibacterial efficacy was assessed using the well diffusion method, and the zone of inhibition was measured at three concentrations: 50, 75, and 100 µg/mL.

**TABLE 1:** The zone of inhibition (mm) for each bacterium at varying concentrations of C2

MDR bacteria	50 µg/mL	75 µg/mL	100 µg/mL
*E. coli*	11 ± 1.0	14 ± 1.3	16 ± 1.1
*S. aureus*	12 ± 1.0	18 ± 1.4	22 ± 1.2
*K. pneumoniae*	15 ± 1.2	16 ± 1.1	21 ± 1.4

The results revealed a concentration-dependent increase in antibacterial activity. At 100 µg/mL, C2 exhibited the highest antibacterial potency, with a maximum zone of inhibition of 22 ± 1.2 mm against *S. aureus*, indicating that this strain was the most susceptible among the tested organisms. Notably, even at 75 µg/mL, *S. aureus* exhibited a zone of inhibition of 18 ± 1.4 mm, underscoring its high sensitivity to the azomethine compound. In contrast, *K. pneumoniae* showed moderate susceptibility, with a maximum zone of 21 ± 1.4 mm at 100 µg/mL, whereas *E. coli* displayed the least sensitivity, producing a 16 ± 1.1 mm inhibition zone at the same concentration (Fig. 5A through C and Table 1).

The visual observations from Fig. 3 align with the quantitative results, showing clear zones of inhibition surrounding the wells filled with azomethine compound C2. The comparative effectiveness of C2 was found to be superior to that of selected commercial antibiotics, which exhibited minimal or no inhibitory effects against the same MDR isolates under identical experimental conditions.

These findings are consistent with previous reports. For example, Ozdal and Gurkok (13) reported that azomethine derivatives possess potent antimicrobial properties due to the presence of the imine (–CH = N–) functional group, which enhances their interaction with bacterial membranes, leading to growth inhibition or cell lysis. Moreover, the higher efficacy observed against *S. aureus* could be attributed to the compound’s ability to disrupt Gram-positive cell walls more efficiently compared to Gram-negative bacteria, which possess an additional outer membrane barrier. The azomethine compound C2 exhibits strong bactericidal properties, particularly against *S. aureus*, suggesting its potential as a promising alternative antibacterial agent, especially in the face of rising antimicrobial resistance.

### Antibacterial activity of titanium dioxide nanoparticles (TiO_2_ NPs)

**Fig 6:**
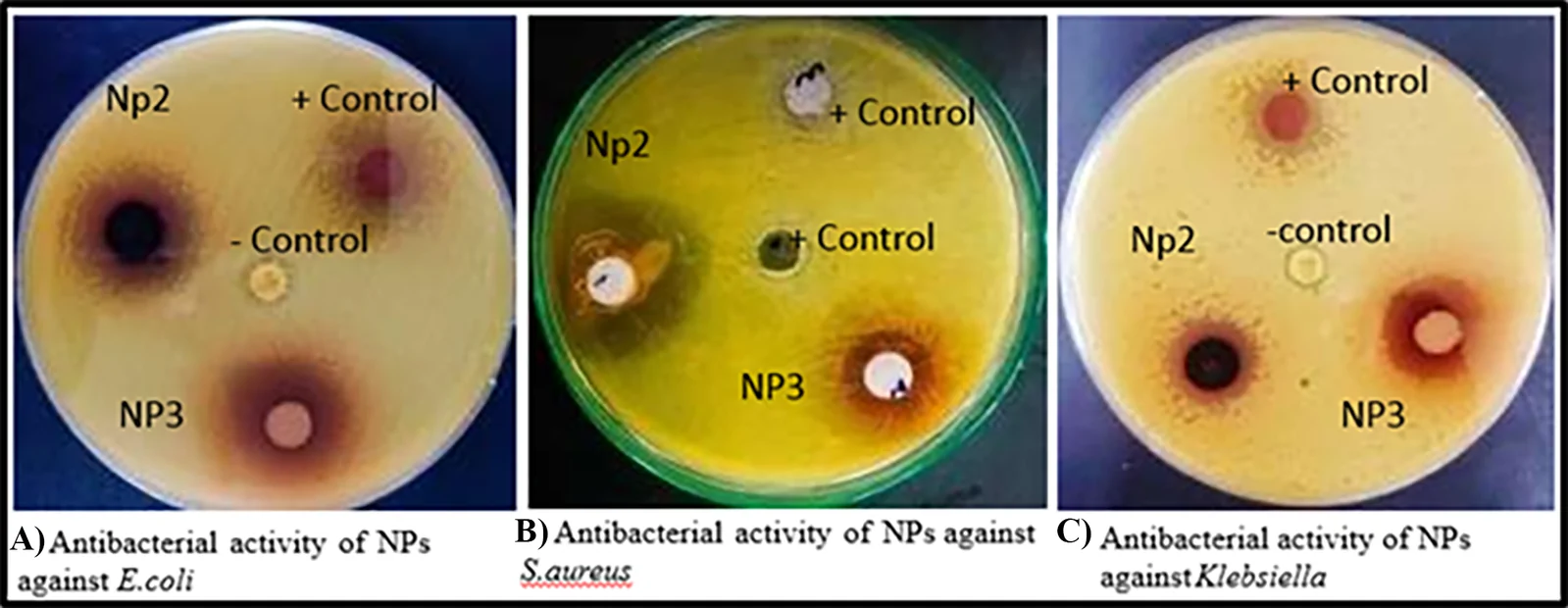
Antibacterial activity of TiO_2_ NPs 100 µL each (NP2 and NP3) against MDR bacterial strains of (A) *E. coli,* (B) *S. aureus,* and (C) *Klebsiella.*

The antibacterial potential of titanium dioxide nanoparticles (TiO_2_ NPs) was evaluated against multidrug-resistant (MDR) bacterial strains, including *E. coli*, *K. pneumoniae*, and *S. aureus*. The nanoparticles were tested in two formulations, labeled NP2 and NP3, at three concentrations: 50, 75, and 100 µg/mL. An antibiotic disc was used as a negative control, while DMSO served as a positive control (Fig. 6A through C and Table 2).

**TABLE 2:** Antibacterial activity of NP2 and NP3 nanoparticle formulations and their zone of inhibition aginst MDR strains

MDR bacteria	NP2 (zone of inhibition in mm), 50 µg	NP3 (zone of inhibition in mm), 75 µg
*E. coli*	23	24
*K. pneumoniae*	18	21
*S. aureus*	19	22

The results revealed that TiO_2_ NPs exerted potent antibacterial effects, showing clear zones of inhibition that increased with nanoparticle concentration. NP2 exhibited superior antibacterial activity compared to NP3 across all tested strains. At 100 µg/mL, the maximum zone of inhibition was observed against *E. coli* (26 mm), followed by *S. aureus* (23 mm) and *K. pneumoniae* (23 mm). NP3 showed slightly lower inhibition zones for the same concentrations, with 24 mm for *E. coli*, 21 mm for *S. aureus*, and 20 mm for *K. pneumoniae*. The antibacterial performance of NP2 was also compared with NP3, the control TiO_2_ nanoparticles synthesized without the Schiff base ligand, to evaluate the role of the azomethine compound in enhancing nanoparticle activity (Fig. 6A and B; Table 2).

*S. aureus* showed considerable sensitivity, with a 23 mm zone of inhibition at the highest concentration, indicating significant bactericidal potential. *K. pneumoniae* was the least sensitive among the tested bacteria, but still showed notable susceptibility to TiO_2_ NPs (Fig. 6A and B; Table 2).

These findings are consistent with earlier reports highlighting the broad-spectrum antimicrobial activity of TiO_2_ nanoparticles. Reports also revealed that, TiO_2_ NPs exhibit effective antibacterial action due to their ability to generate reactive oxygen species (ROS) under ambient or UV conditions, which can damage bacterial cell membranes, proteins, and DNA. Similarly, the work of Foster et al. (14) emphasized that TiO_2_ nanoparticles disrupt microbial cell wall integrity and interfere with metabolic functions, leading to bacterial death. Moreover, the increasing zone of inhibition with concentration supports the dose-dependent antibacterial nature of TiO_2_ nanoparticles, as previously reported by Raghupathi et al. (15), who observed enhanced antimicrobial activity with higher NP surface availability and reduced particle size.

### Determination of MIC and MBC of nanoparticles and azomethine compound

**Fig 7:**
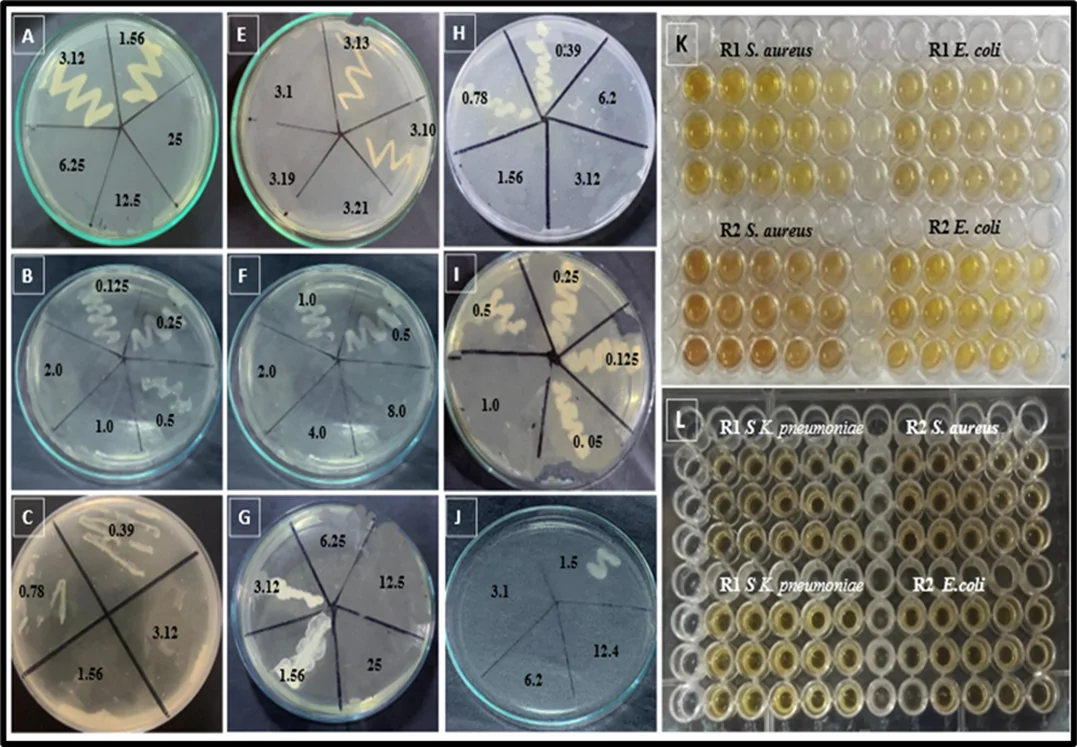
The *in vitro* antibacterial activity (MBC and MIC) of the synthesized nanoparticles against clinically relevant MDR bacterial pathogens was evaluated at varying concentrations. Panels **A–C** depict the antibacterial activity against *S. aureus*, where panel **A** corresponds to the C2 nanoparticle, panel **B** corresponds to NP2, and panel **C** corresponds to NP3. Panels **E–G** illustrate antibacterial activity against *E. coli* using the same nanoparticles (C2 [**E**], NP2 [**F**], and NP3 [**G**]). Panels **H–J** represent antibacterial activity against *K. pneumoniae* (C2 [**H**], NP2 [**I**], and NP3 [**J**]). Numerical values within each sector denote the tested concentrations (µg/mL), panels K and L represents the microwell plate display MIC with varying turbidity across replicates for MDR strains.

The minimum inhibitory concentration (MIC) and minimum bactericidal concentration (MBC) of titanium dioxide nanoparticles (NP2 and NP3) and the azomethine compound (C2) were evaluated against three multidrug-resistant (MDR) bacterial strains *S. aureus*, *E. coli*, and *K. pneumoniae* (Table 3 and Fig. 7). The MIC represents the lowest concentration of the compound that inhibits visible bacterial growth, while the MBC is the lowest concentration that completely eradicates the bacteria.

**TABLE 3:** MIC and MBC values (µg/mL) of nanoparticles and azomethine compound (C2)

Nanoparticle	*S. aureus*		*E. coli*		*K. pneumoniae*	
	MIC	MBC	MIC	MBC	MIC	MBC
C2	3.13 ± 0.11	6.25 ± 0.15	1.5 ± 0.10	3.13 ± 0.10	0.75 ± 0.05	1.5 ± 0.08
NP2	0.5 ± 0.04	1.0 ± 0.05	2.0 ± 0.07	2.0 ± 0.07	0.5 ± 0.04	1.0 ± 0.05
NP3	0.75 ± 0.05	1.5 ± 0.10	3.13 ± 0.11	6.25 ± 0.15	1.5 ± 0.06	3.13 ± 0.08

The results, presented in Table 3, reveal that all tested agents exhibited notable antibacterial effects, with varying degrees of potency depending on the nanoparticle formulation and bacterial strain. Among the three agents, NP2 exhibited the strongest antibacterial activity, showing the lowest MIC and MBC values across all tested strains. For instance, NP2 had MIC and MBC values of 0.5 ± 0.04 and 1.0 ± 0.05 µg/mL, respectively, against *S. aureus* and *K. pneumoniae*, and 2.0 ± 0.07 µg/mL against *E. coli*. In comparison, NP3 showed higher MIC/MBC values, indicating slightly reduced efficacy (Fig. 7 and Table 3).

The azomethine compound C2 also showed effective antibacterial action, particularly against *K. pneumoniae*, with MIC values of 0.75 ± 0.05 µg/mL and MBC values of 1.5 ± 0.08 µg/mL. Against *E. coli*, C2 showed MIC values of 1.5 ± 0.10 µg/mL and MBC values of 3.13 ± 0.10 µg/mL, while the highest values were observed for *S. aureus* (MIC 3.13 ± 0.11 µg/mL, MBC 6.25 ± 0.15 µg/mL), indicating comparatively lower sensitivity (Fig. 7 and Table 3).

These results align with previous findings indicating that TiO_2_ nanoparticles possess strong antimicrobial properties due to their ability to generate reactive oxygen species (ROS) and disrupt microbial membranes (14). The antibacterial activity observed in this study may be partly attributed to the photocatalytic nature of TiO_2_ nanoparticles, which are known to generate reactive oxygen species (ROS), such as hydroxyl radicals and superoxide ions, under UV irradiation. These ROS can induce oxidative stress in bacterial cells, leading to damage of cellular membranes, proteins, and nucleic acids, ultimately resulting in bacterial death. Although the UV–visible absorption characteristics of the synthesized TiO_2_ nanoparticles suggest their potential for ROS generation, direct experimental verification of ROS production was not performed in the present study. Therefore, future investigations involving ROS detection assays (such as DCFH-DA fluorescence or electron spin resonance analysis) will be necessary to further elucidate the precise antibacterial mechanism of the synthesized nanoparticles.

Furthermore, the enhanced activity of NP2 may be attributed to its smaller particle size and better surface reactivity compared to NP3, leading to improved interaction with bacterial cells. The antibacterial effect of azomethine compounds has also been previously reported. According to Ozdal and Gurkok (13), azomethine-based molecules can interfere with microbial enzyme systems and cellular respiration, contributing to their antimicrobial potency. The relatively lower MIC values observed against *K. pneumoniae* in this study support those earlier findings. Overall, these results indicate that both TiO_2_ NPs, particularly NP2, and azomethine compound C2 exhibit significant antibacterial activity and could serve as promising agents for combating multidrug-resistant bacteria.

### Conclusion

This study successfully demonstrated the catalytic synthesis and antibacterial potential of Schiff base-functionalized TiO_2_ nanoparticles. Structural and spectral analyses confirmed the formation of well-defined nanoparticles with enhanced physicochemical properties. Both the azomethine compound (C2) and TiO_2_ NPs, particularly NP2, exhibited strong antibacterial activity against multidrug-resistant *E. coli*, *S. aureus*, and *K. pneumoniae*. NP2 showed the lowest MIC and MBC values, indicating superior bactericidal efficiency. These results suggest that Schiff base-modified TiO_2_ nanoparticles offer a promising strategy for combating antibiotic-resistant pathogens and warrant further investigation for biomedical applications.
